# Are Your kidneys ok? Detect early to protect kidney health

**DOI:** 10.1590/2175-8239-JBN-2025-0106en

**Published:** 2025-07-07

**Authors:** Joseph A. Vassalotti, Anna Francis, Augusto Cesar Soares Dos Santos, Ricardo Correa-Rotter, Dina Abdellatif, Li-Li Hsiao, Stefanos Roumeliotis, Agnes Haris, Latha A. Kumaraswami, Siu-Fai Lui, Alessandro Balducci, Vassilios Liakopoulos

**Affiliations:** 1 Mount Sinai Hospital, Department of Medicine-Renal Medicine, NY, New York, United States.; 2 Queensland Children’s Hospital, Department of Nephrology, South Brisbane, Queensland, Australia.; 3 Universidade Federal de Minas Gerais, Hospital das Clínicas (EBSERH), Belo Horizonte, MG, Brazil.; 4 Faculdade de Ciências Médicas de Minas Gerais, Belo Horizonte, MG, Brazil.; 5 Instituto Nacional de Ciencias Médicas y Nutrición Salvador Zubirán, Mexico City, Mexico.; 6 Cairo University, Cairo University Hospital, Department of Nephrology, Cairo, Egypt.; 7 Harvard Medical School, Brigham and Women’s Hospital, Renal Division, Department of Medicine, Boston, MA, USA.; 8 Aristotle University of Thessaloniki, Medical School, AHEPA University Hospital, Second Department of Nephrology, Thessaloniki, Greece.; 9 Péterfy Hospital, Nephrology Department, Budapest, Hungary.; 10 Tamilnad Kidney Research (TANKER) Foundation, Chennai, India.; 11 The Chinese University of Hong Kong, Jockey Club School of Public Health and Primary Care, Division of Health System, Policy and Management, Hong Kong.; 12 Italian Kidney Foundation, Rome, Italy.

**Keywords:** Mass Screening, Case Finding, Renal Insufficiency, Chronic, Albuminuria, Proteinuria, Disease Prevention

## Abstract

Early identification of kidney disease can protect kidney health, prevent kidney disease progression and related complications, reduce cardiovascular disease risk, and decrease mortality. We must ask “*Are your kidneys ok*?” using serum creatinine to estimate kidney function and urine albumin to assess for kidney and endothelial damage. Evaluation for causes and risk factors for chronic kidney disease (CKD) includes testing for diabetes and measurement of blood pressure and body mass index. On this World Kidney Day, we emphasize that case-finding in high-risk populations or even population level screening can decrease the burden of kidney disease globally. Early-stage CKD is asymptomatic, simple to test for, and recent paradigm shifting CKD treatments such as sodium glucose co-transporter-2 inhibitors dramatically improve outcomes and favor the cost-benefit analysis for screening or case-finding programs. Despite this, numerous barriers exist, including resource allocation, healthcare funding, healthcare infrastructure, and awareness of kidney disease among healthcare professionals and the general population. Coordinated efforts by major kidney non-governmental organizations to prioritise the kidney health agenda for governments and align early detection efforts with other current programs will maximise efficiency.

## Introduction

Timely treatment is the primary strategy to protect kidney health, prevent kidney disease progression and related complications, reduce cardiovascular disease risk, and prevent premature kidney-related and cardiovascular mortality^1,2,3^. International population assessments show low awareness, low detection of kidney disease, and substantial gaps in treatment^2^. People with kidney failure universally express their desire for having been diagnosed early in their disease trajectory to allow more time for educational, lifestyle and pharmacologic interventions^4^. Therefore, increasing knowledge and implementing sustainable solutions for early detection of kidney disease to protect kidney health are public health priorities^2,3^.

### Epidemiology and Complications of Kidney Disease

CKD (chronic kidney disease) is a prevalent condition, affecting 10% of the world’s population or over 700 million people^5^. Almost 80% of the population with CKD reside in low-income countries (LICs) and lower-middle-income countries (LMICs), with approximately 1/3 of the known affected population living in China and India alone^5,6^. Prevalence of CKD increased by 33% between 1990 and 2017^5^. Increasing prevalence of CKD is driven by population growth, aging, and the obesity epidemic, which increases the prevalence of two major risk factors for CKD: type-2 diabetes (T2DM) and hypertension. In addition, risk factors for CKD beyond cardiometabolic conditions contribute to the rising burden of kidney disease, including social deprivation, pregnancy-related acute kidney injury (AKI), preterm birth, and increasing environmental threats (infections, toxins, climate change, air pollution)^5,7^. These threats disproportionately affect people in LICs and LMICs^8^.

Undetected and untreated CKD is more likely to progress to kidney failure and cause premature morbidity and mortality. Globally, more people died in 2019 of cardiovascular disease (CVD) attributed to reduced kidney function (1.7 million people) than kidney disease alone (1.4 million)^5^. CKD is expected to rise to the 5^th^ most common cause of years of life lost by 2040, surpassing type 2 diabetes, Alzheimer’s disease and road injuries^9^. The rising mortality of kidney disease is remarkable in contrast to other non-communicable diseases (NCDs) such as CVD, stroke, and respiratory disease, which are projected to experience a decline in mortality^8^. Even in early stage CKD, multi-system morbidity decreases quality of life. In particular, mild cognitive impairment is associated with early stage CKD and it is possible that early CKD detection and treatment could slow cognitive decline and reduce the risk of dementia^10^. CKD in children has profound additional effects, threatening growth and cognitive development and having lifelong health and quality of life implications^11,12^. The number of people on kidney failure replacement therapy (KFRT) – dialysis and transplantation – is anticipated to more than double to 5.4 million from 2010 to 2030^13,14^. KFRT, especially hemodialysis, is unavailable or unaffordable to many in LICs and LMICs, contributing to millions of deaths annually. LICs and LMICs comprise 48% of the global population but account for only 7% of the treated kidney failure population^15^.

## Who is at Risk of Kidney Disease?

Testing people at high-risk of kidney disease (case-finding) limits potential harms and false-positive test results compared to screening the general population, wich should only be considered in high-income countries (HICs). Limiting testing to those at increased risk of CKD would still capture a large proportion of the global population. Moreover, targeted case-finding in patients at high risk of CKD, is not optimally performed even in HICs. About 1 in 3 people worldwide have diabetes and/or hypertension. There is a bidirectional relationship between cardiovascular disease and CKD, with each increasing the risk of the other. The American Heart Association and European Society of Cardiology call for testing those with cardiovascular disease for CKD, as part of routine cardiovascular assessments^1,16^.

**Figure 1:**
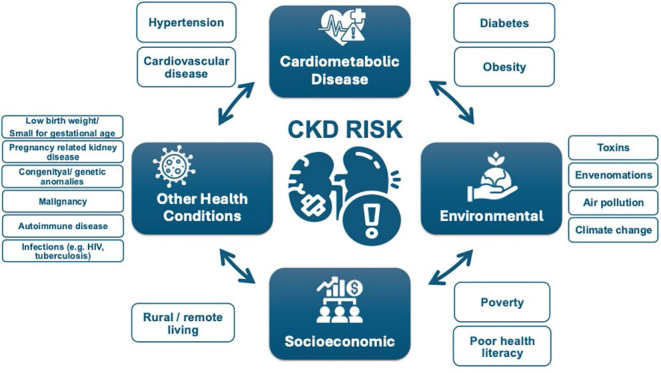
Risk factors for CKD.

Other CKD risk factors include family history of kidney disease (e.g. APOL1-mediated kidney disease common in people of West African ancestry), prior AKI, pregnancy-related kidney disease (e.g. pre-eclampsia), malignancy, autoimmune disorders (systemic lupus erythematosus, vasculitis), individuals born with low birth weight or pre-term, obstructive uropathy, recurrent kidney stones, and congenital anomalies of the kidney and urinary tract (CAKUT) (see Figure 1)^3^. The social determinants of health strongly affect CKD risk, both for individuals and at country level. In the LICs and LMICs, heat stress is thought to cause CKD of unknown etiology in agricultural workers, an increasingly recognized major global cause of CKD^17^. In addition, envenomations, environmental toxins, traditional medicines and infections (viral hepatitis B or C, HIV, and parasites) deserve consideration as risk groups, especially in endemic areas^18,19^.

## How can we check Kidney Health?

Conceptually, there are three levels of CKD prevention. Primary prevention reduces the incidence of CKD by treating risk factors; secondary prevention reduces progression and complications in those with detected CKD; and tertiary prevention improves outcomes in those with kidney failure by improving management, such as improved vaccination or optimal dialysis delivery^20^. Primary and secondary prevention strategies can utilise the 8 golden rules for kidney health promotion; healthy diet, adequate hydration, physical activity, blood pressure monitoring and control, glycaemic monitoring and control, avoidance of nicotine, avoidance of regular use of non-steroidal anti-inflammatory drugs, and targeted testing for those with risk factors^21^. Five of these are identical to ‘Life’s Essential 8’ rules for improving and maintaining cardiovascular health, which also include healthy weight, adequate sleep, and lipid management^22^. Early detection focuses on secondary CKD prevention, which involves protecting kidney health and reducing cardiovascular risk.

### Are Your Kidneys Okay?

Globally, early detection of CKD is rare, haphazard, and even less likely to occur in LICs or LMICs. Currently, only three countries have a national program to actively test at-risk populations for CKD, and a further 17 countries actively test at-risk populations during routine health screening^23^. Even in HICs, albuminuria is not assessed in over half of people with T2DM and/or hypertension^24,25,26^. Startlingly, in those with documented reduced kidney function, a diagnosis of CKD is often missing. A study in HICs showed absence of CKD diagnosis among 62-96% of the population with laboratory evidence of CKD stage G3^27^.

**Figure 2:**
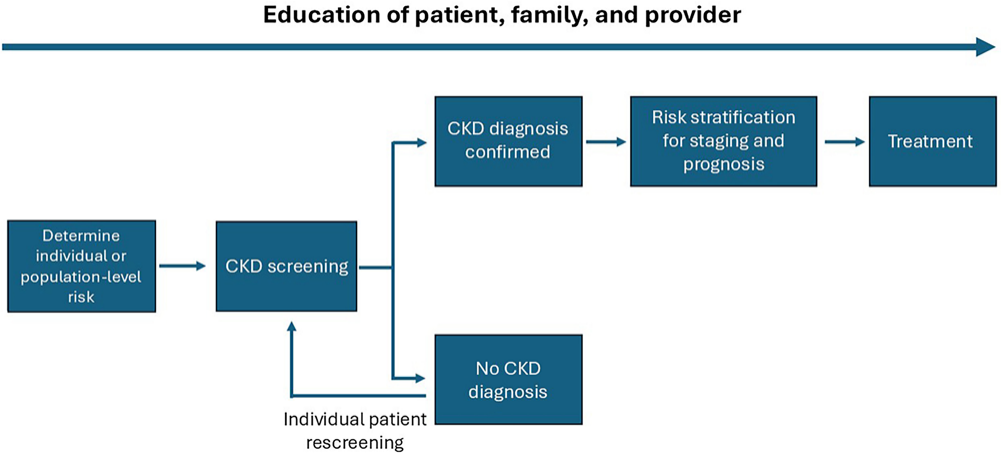
Conceptual framework of a CKD testing, risk stratification, and treatment program, see reference^30^.

We recommend that healthcare professionals perform the following tests for all risk groups to assess kidney health (Figure 2):

a) Blood pressure measurements, as hypertension is the most prevalent risk factor for kidney disease worldwide^3,28,29^.
b) Body mass index (BMI), since obesity is epidemiologically associated with CKD risk indirectly through T2DM and hypertension and as an independent risk factor. Visceral adiposity contributes to monocyte microinflammation and cardiometabolic kidney risk^3,28,29^.
c) Glycosylated haemoglobin or fasting blood sugar or random glucose, as T2DM is a common risk factor for kidney disease^3,28,29^.
d) Evaluating kidney function by using serum creatinine to estimate GFR (eGFR), is recommended in all settings^3^. GFR should be estimated with a validated, race-free equation appropriate for the country or region and age group^3^. In general, eGFR < 60 mL/min/1.73m^2^ is the threshold for CKD in adults and children, although a threshold of < 90 mL/min/1.73m^2^ can be flagged as “low” in children and adolescents over the age of 2 years^3^. A limitation of creatinine-based eGFR is that creatinine is also a marker of nutrition and muscle mass. Therefore, states of malnutrition and frailty overestimate kidney function^3,30^. Thus, eGFR combining serum creatinine and cystatin C is generally more accurate than either biomarker alone in most clinical contexts. However, the feasibility of cystatin C use is mainly limited to HICs because of assay availability and cost of creatinine^3,30,31^.
e) Testing for kidney damage (albuminuria). In adults and children, a first morning urine sample is preferred for assessing albuminuria^3^. In adults, quantitative urinary albumin-creatinine ratio (uACR) is preferred as the most sensitive test^3^. Importantly, urinary albumin is in the process of being standardized analytically, which should ultimately facilitate worldwide uACR standardization^32^. In children, both protein-creatinine ratio (uPCR) and uACR should be tested in order to assess tubular proteinuria^3^. Semiquantitative albuminuria testing allows for flexibility for point-of-care or home-based testing^33^. Semiquantitative or qualitative screening tests should be positive in >85% of individuals with quantitative uACR 30 mg/g or more to be useful^34^. In resource-constrained settings, urine protein dipstick testing may be used with a threshold of +2 proteinuria or greater to reduce false positive results for repeat confirmatory testing^35^.

In specific populations, the following can be considered:

f) Testing for haematuria, which has been known as the forgotten risk factor in recent clinical practice guidelines, is particularly important for those at risk for glomerular disease, particularly IgA nephropathy^36^.
g) Baseline imaging in groups with signs or symptoms of structural abnormalities (e.g. pain and haematuria) to evaluate for kidney masses, cysts, stones, hydronephrosis or urinary retention is important. Antenatal ultrasound can detect hydronephrosis and other CAKUT.
h) With increasing availability of genetic testing, family cascade CKD testing is indicated when there is a known genetic risk of kidney disease^37^.
i) In those who have an occupational risk of developing kidney disease, kidney testing should be offered as part of occupational health programs.
j) Those who donate kidneys should be included in a post-donation surveillance program to assess kidney health over the long-term^38^.

## Potential Benefits of Early Detection

Screening for CKD fits with many of the World Health Organisation’s Wilson-Jungner principles. Early stage CKD is asymptomatic and effective treatments, including lifestyle modification, interdisciplinary care, and pharmacologic interventions, are established^2,3,30,35^. WHO essential medicines that improve CKD outcomes, including ACE inhibitors, angiotensin receptor blockers, statins, and sodium glucose co-transporter-2 inhibitors (SGLT2i), should be widely available^2,39^. SGLT2i alone are estimated to decrease the risk of CKD progression by 37% in people with and without diabetes^40^. For a 50-year old person with albuminuria and non-diabetic CKD, this could extend the period of healthy kidney function from 9.6 years to 17 years^41^. These essential medicines reduce progression to more advanced CKD stages and limit cardiovascular hospitalization to provide short-term cost-effectiveness, especially in LICs. Where available and affordable, the range of new paradigm-shifting medications to slow CKD progression also includes glucagon-like peptide-1 receptor antagonists, non-steroidal mineralocorticoid receptor antagonists, endothelin receptor antagonists, and specific disease-modifying drugs (e.g. complement-inhibitors) that herald an exciting new era for nephrology.

Considering the significant healthcare costs associated with CKD, particularly hospitalization and kidney failure, effective preventive measures offer clear economic benefits for both high- and low-income countries. CKD confers enormous costs to the individual, their families, healthcare systems, and governments worldwide. In the United States, CKD costs Medicare over US$ 85 billion annually^13^. In many high- and middle-income countries, 2–4% of the health budget is spent on kidney failure care alone. In Europe, healthcare costs associated with CKD are higher than those associated with cancer or diabetes^42^. Reducing the burden of kidney care worldwide will also have profound environmental effects, as it will save water and reduce plastic waste, especially associated with dialysis^43^. On an individual level, CKD costs are frequently catastrophic, particularly in LICs and LMICs, where the individual largely bears the burden of payment. Only 13% of LICs and 19% of LMICs cover the costs of KFRT for adults^15^. CKD causes 188 million people in low and lower-middle-income countries annually to be faced with catastrophic healthcare expenditures^44^.

The most widely cited and studied incremental cost effectiveness ratio (ICER) threshold to assess screening is US$ <50,000 per quality-adjusted life year (QALY)^45^. If the prevalence of CKD is high, a population-wide screening strategy should be considered in HIC^33,46^. For example, in the United States, a recent Markov simulation model of population-wide screening for CKD, which included appropriate SGLT2i treatment added to standard of care ACE inhibitors or angiotensin receptor blockers for adults age 35 to 75 years old with albuminuria, concluded that screening to identify CKD would be cost-effective^46^. In addition, an analysis of a home-based general population semiquantitative albuminuria screening in Holland was also found to be cost effective^33^. Case finding to detect CKD in higher risk groups rather than mass or general population screening will reduce costs and other harms whilst increasing the true positive rate of the screening tests^3,35,45^. An alternate ICER threshold proposed by WHO is <1–3 times the ratio of the gross domestic product per capita income per QALY, which can be used to assess case finding approaches in LIC and LMIC^45^. The recommended tests for detecting kidney disease are low-cost and minimally invasive, facilitating their administration across diverse settings. Basic testing of eGFR and urinary ACR are widely available, and using urine dipstick testing where quantitative proteinuria testing is unavailable or unaffordable will drastically reduce testing costs^31^.

If coupled with effective intervention, early identification of people with kidney disease will benefit the individual, the health care system, governments, and the economy^44^. Health and quality of life benefits for the individual would lead to improved productivity, especially in the young who have more working years ahead, and to developmental/educational improvements in children and young adults. Individuals would face less catastrophic health expenses, gov­ernments and healthcare systems will save money not only on CKD care, but also on cardiovascular disease costs, and economies will benefit from higher worker participation. This is especially crucial for low-income countries where the burden of CKD is where gov­ernments and individuals cannot afford kidney care.

## Challenges and Solutions for Implementation

Structural barriers to widespread identification and treatment of people with CKD include cost, test reliability, and lack of health information systems to track CKD burden. These are underpinned by a lack of relevant government and healthcare policies, low healthcare professional knowledge and implementation, low perceived kidney disease risk in the general population, and low patient CKD awareness. Solutions for implementing effective interventions include tying CKD identification to existing screening programs, educating the public and primary care professionals, and leveraging non-governmental organization (NGO) joint advocacy programs to focus health policy agendas on kidney disease. Any solutions must balance the potential benefits and harms of screening and case-finding programs. Ethical implications for consideration include the availability of resources (such as health care workers and medicines), the affordability of testing and treatment, false positives or negatives, and anxiety for patients and their families^47^.

Screening and case-finding programs require workforce capacity, health information systems, reliable testing equipment and equitable access to medical care, medicines, vaccines and medical technologies. Primary care is at the front line of the battle to protect kidney health, particularly in low and low-to-middle-income countries. The tiny nephrology workforce, with a median global prevalence of 11.8 nephrologists per million population and an 80-fold discrepancy between LICs and HICs, is inadequate to detect and manage the vast majority of CKD^23^. As for other chronic diseases, primary care clinicians and other frontline health workers are foundational to early detection of CKD^48^. Testing must be affordable, simple, and practical, with point-of-care creatinine testing and urine dipsticks useful in resource-limited settings^31^. Educational efforts directed at primary care clinicians are key to integrating CKD detection into routine care, despite constrained time and resources^49,50,51^. Automated clinical decision support could leverage electronic health records to identify people with CKD or at high-risk of CKD and recommend appropriate actions to clinicians (Figure 2).

Currently, few countries have CKD registries, limiting our ability to highlight the disease burden to governments. Knowledge of CKD burden helps prioritize kidney health needs, which should then progressively expand to encompass the full spectrum of kidney care^52^. A global survey revealed that only a quarter of the countries (41/162) had a nation-specific CKD strategy and fewer than a third (48/162) recognized CKD as a public health priority^23^. WHO’s recognition of CKD as a major driver of NCD mortality would be effective in increasing awareness, improving local surveillance and monitoring to implement clinical practice guidelines, and improving resource allocation^2^.

Programs for the early detection of CKD will require extensive coordination and engagement of stakeholders, including governments, health systems, and insurers. International and national kidney organisations, such as the International Society of Nephrology (ISN), already advocate to the WHO and individual governments for the prioritisation of kidney disease. We must continue this work, collaborating to streamline early detection program planning and implementation. The integration with existing community programs (e.g. cardiovascular disease prevention) in LMICs and HICs can decrease cost and maximize efficiencies. Such programs will need to be adapted to the local context and can be held in a variety of settings, such as individual healthcare practices, hospitals, regional or national healthcare facilities or as outreach to rural communities. Depending on local regulations and resources, screening and case-finding can also take place outside of medical settings such as town halls, churches, or markets. Volunteers in the community can also assist with community-based screening and case-finding efforts.

In conjunction with refocusing the clinical practice of health care professionals on the timely detection of CKD, we must focus on educating the general population about perceived risk and on health promotion activities and educational programs to raise awareness and empower patients. Awareness of kidney disease is poor, with 9 out of 10 people with CKD not knowing that they are affected^53^. Kidney disease is poorly covered in the lay press, and an analysis showed that kidney disease was 11-times less-represented compared to the actual cause of death^54^. A number of national and international organizations have developed quizzes on risk of kidney disease for the general public. This was supported by a regional study that showed that socially vulnerable patients with hypertension do not understand their kidney risks^21,55,56,57^. Online and in-person education for healthcare professionals can improve consumer health literacy. Patient activation, engagement, and shared decision-making are down­stream impacts of awareness. CKD awareness is nuanced, including detection and risk stratification to inform and empower, rather than scare, regarding the timing and extent of interventions (see Chart 1)^4,27,57^. Finding the right balance will optimize patient, family, and caregiver self-efficacy and engagement.

**Chart 1:** Are your kidneys okay? personal perspectives on CKD awareness, detection and treatment from the literature ^4,57^

I actually didn’t fully understand why nobody had actually given me the full information of what I had in a way that I could kind of go, ‘Well this is what I’ve got [CKD], and this is why I’ve got it.’
They [the clinicians] can answer those [kidney health] questions, ... but it’s all very jargonistic.
I didn’t know what it [CKD] meant so I couldn’t really share it with other people.
I may not know what my [kidney health] numbers are, but I do know what the tests are, and I do know that I’ve had them done before.
Well, let me put this way: I’m now well aware of the significance of the kidneys and about what the issues are here. And I would definitely consider... When I go to the doctor, I would say to him, “Now, listen. You did the blood tests. But how are my kidneys doing? What are the numbers?
I know that they have done urine tests in the past, and I know protein and sugar was in my urine.
I went from never taking a tablet to taking 22 tablets. What is going on here? I didn’t know what they were. But I just number them and that did help me a lot because I realized what was going on but some of them, every time I went there [to see the doctor], I’d get another tablet. I knew that I had to take it because they knew what they were doing, the doctors that I went to see.
This [CKD] is something new, so immediately I was like, just another thing to be concerned about. But then I felt kind of empowered, and like I really do want to get ahead of this thing. I feel like I do want to have a conversation with my primary care physician.
What I would be mostly interested in is what is happening, why is it happening, and what can I do to slow it [CKD] down?

## Conclusion: A Call to Action

We call on all healthcare professionals to check the kidney health of their patients at risk of kidney disease. In tandem, we must work with public health organizations to improve the general population’s perceived risk of kidney disease and empower people at risk to seek kidney health checks. To ensure this change can be delivered, we must work with healthcare systems, governments, and the WHO to prioritize kidney disease and create effective and efficient early detection programs for kidney disease. Only then will the paradigm-shifting benefits of lifestyle change and pharmacologic treatments translate to better kidney and overall health for people all around the world.

## Data Availability

There is no new data associated with this article.
