# Supplementary material for: Are Your kidneys ok? Detect early to protect kidney health
Source: J Bras Nefrol. 2025 Jul 7;47(4):e20250106. doi: 10.1590/2175-8239-JBN-2025-0106en (PMC12233814; doi:10.1590/2175-8239-JBN-2025-0106en)
Supplement: Translation

# Translation: Seus rins estão saudáveis? Faça um diagnóstico precoce para proteger a saúde renal

**DOI:** 10.1590/2175-8239-JBN-2025-0106pt

**Authors:** Joseph A. Vassalotti, Anna Francis, Augusto Cesar Soares Dos Santos, Ricardo Correa-Rotter, Dina Abdellatif, Li-Li Hsiao, Stefanos Roumeliotis, Agnes Haris, Latha A. Kumaraswami, Siu-Fai Lui, Alessandro Balducci, Vassilios Liakopoulos

**Affiliations:** 1 Mount Sinai Hospital, Department of Medicine-Renal Medicine, NY, New York, Estados Unidos.; 2 Queensland Children’s Hospital, Department of Nephrology, South Brisbane, Queensland, Austrália.; 3 Universidade Federal de Minas Gerais, Hospital das Clínicas (EBSERH), Belo Horizonte, MG, Brasil.; 4 Faculdade de Ciências Médicas de Minas Gerais, Belo Horizonte, MG, Brasil.; 5 Instituto Nacional de Ciencias Médicas y Nutrición Salvador Zubirán, Mexico City, México.; 6 Cairo University, Cairo University Hospital, Department of Nephrology, Cairo, Egito.; 7 Harvard Medical School, Brigham and Women’s Hospital, Renal Division, Department of Medicine, Boston, MA, EUA.; 8 Aristotle University of Thessaloniki, Medical School, AHEPA University Hospital, Second Department of Nephrology, Thessaloniki, Grécia.; 9 Péterfy Hospital, Nephrology Department, Budapest, Hungria.; 10 Tamilnad Kidney Research (TANKER) Foundation, Chennai, Índia.; 11 The Chinese University of Hong Kong, Jockey Club School of Public Health and Primary Care, Division of Health System, Policy and Management, Hong Kong.; 12 Italian Kidney Foundation, Rome, Itália.

**Keywords:** Programas de Rastreamento, Detecção de casos, Insuficiência Renal Crônica, Albuminúria, Proteinúria, Prevenção de Doenças

## Abstract

A identificação precoce da doença renal pode proteger a saúde dos rins, prevenir a progressão da doença e complicações relacionadas, reduzir o risco de doenças cardiovasculares e diminuir a mortalidade. Devemos questionar “*Seus rins estão saudáveis*?” utilizando creatinina sérica para estimar a função renal e albumina urinária para avaliar lesões renais e endoteliais. A avaliação das causas e dos fatores de risco para a doença renal crônica (DRC) inclui testes para diabetes e medição da pressão arterial e do índice de massa corporal. Neste Dia Mundial do Rim, enfatizamos que a detecção de casos em populações de alto risco, ou mesmo a triagem em nível populacional, pode reduzir o ônus da doença renal globalmente. A DRC em estágio inicial é assintomática, facilmente diagnosticável e os recentes tratamentos, que representam uma mudança de paradigma, como inibidores do cotransportador de sódio-glicose tipo 2, promovem uma melhora significativa nos desfechos da doença e favorecem a análise custo-benefício para programas de triagem ou detecção de casos. Apesar disso, existem diversos obstáculos, incluindo a alocação de recursos, o financiamento da saúde, a infraestrutura dos serviços de saúde e a conscientização sobre a doença renal entre profissionais da saúde e a população em geral. Esforços coordenados pelas principais organizações não governamentais dedicadas a doenças renais, com o objetivo de priorizar a agenda de saúde renal junto aos governos e alinhar as iniciativas de detecção precoce com outros programas atuais, irão maximizar a eficiência.

## Introdução

O tratamento oportuno constitui a principal estratégia para proteger a saúde renal, prevenir a progressão da doença renal e complicações relacionadas, reduzir o risco de doenças cardiovasculares e prevenir a mortalidade cardiovascular e renal prematura^1,2,3^. Avaliações populacionais internacionais revelam um baixo nível de conscientização, baixa detecção de doenças renais e lacunas substanciais no tratamento. Indivíduos com insuficiência renal expressam universalmente o desejo de ter sido diagnosticados precocemente ao longo do curso da doença, de modo a dispor de mais tempo para receber intervenções educacionais, farmacológicas e relacionadas ao estilo de vida^4^. Portanto, ampliar o conhecimento e implementar soluções sustentáveis para a detecção precoce da doença renal, a fim de proteger a saúde dos rins, são prioridades de saúde pública^2,3^.

### Epidemiologia e Complicações da Doença Renal

A doença renal crônica (DRC) é uma condição prevalente, que afeta 10% da população mundial, o que equivale a mais de 700 milhões de pessoas^5^. Quase 80% da população com DRC reside em países de baixa renda (LIC, do inglês *low-income countries*) e países de renda média-baixa (LMIC, do inglês *lower-middle income countries*), com aproximadamente 1/3 da população afetada conhecida concentrando-se apenas na China e na Índia^5,6^. A prevalência de DRC aumentou 33% entre 1990 e 2017^5^. Esse aumento é impulsionado pelo crescimento e envelhecimento populacionais e pela epidemia da obesidade, que elevam a prevalência de dois importantes fatores de risco para a DRC: diabetes tipo 2 (DM2) e hipertensão. Além das condições cardiometabólicas, outros fatores de risco para DRC contribuem para o aumento do ônus da doença renal, incluindo privação social, injúria renal aguda (IRA) relacionada à gestação, parto prematuro e ameaças ambientais crescentes (infecções, toxinas, mudanças climáticas, poluição atmosférica)^5,7^. Essas ameaças atingem de maneira desproporcional as populações dos LIC e LMIC^8^.

A DRC não detectada e não tratada apresenta maior probabilidade de progredir para insuficiência renal e provocar morbidade e mortalidade prematuras. Mundialmente, mais pessoas foram a óbito em 2019 por doença cardiovascular (DCV) atribuída à redução da função renal (1,7 milhão de pessoas) do que pela doença renal isolada (1,4 milhão)^5^. Estima-se que a DRC se torne a quinta causa mais comum de anos de vida perdidos até 2040, superando o diabetes tipo 2, a doença de Alzheimer e os acidentes de trânsito^9^. O aumento da mortalidade por doença renal é notável, em contraste com outras doenças não transmissíveis (DNTs), como DCV, acidente vascular cerebral e doenças respiratórias, que, segundo as projeções, sofrerão um declínio na mortalidade^8^. Mesmo nos estágios iniciais da DRC, a morbidade multissistêmica reduz a qualidade de vida. Em particular, o comprometimento cognitivo leve está associado ao estágio inicial da DRC e é possível que a detecção e o tratamento precoces da doença possam retardar o declínio cognitivo e reduzir o risco de demência^10^. A DRC em crianças apresenta profundos efeitos adicionais, comprometendo o crescimento e o desenvolvimento cognitivo, além de ter implicações para a saúde e a qualidade de vida ao longo da vida^11,12^. Espera-se que o número de pessoas em terapia renal substitutiva (TRS) – diálise e transplante – mais do que duplique, chegando a 5,4 milhões entre 2010 e 2030^13,14^. A TRS, especialmente a hemodiálise, não está disponível ou é financeiramente inviável para grande parte da população em LIC e LMIC, contribuindo para milhões de óbitos anualmente. Os LIC e os LMIC compreendem 48% da população global, mas representam somente 7% da população com insuficiência renal tratada^15^.

## Quem está em Risco de Desenvolver Doença Renal?

A testagem de pessoas com alto risco para doença renal (detecção de casos) limita os potenciais danos e os resultados de testes falso-positivos em comparação com a triagem da população em geral, o que só deve ser considerado em países de alta renda (HICs, do inglês *high-income countries*). Restringir os testes àqueles que têm maior risco de desenvolver DRC ainda abrangeria uma grande parte da população global. Além disso, a detecção de casos específicos em pacientes com alto risco para DRC não é realizada de forma ideal, mesmo em HICs. Aproximadamente 1 em cada 3 pessoas no mundo tem diabetes e/ou hipertensão. Existe uma relação bidirecional entre a doença cardiovascular e a DRC, em que uma aumenta o risco da outra. A *American Heart Association* e a *European Society of Cardiology* recomendam que as pessoas com doenças cardiovasculares sejam testadas para a DRC, como parte das avaliações cardiovasculares de rotina^1,16^.

Outros fatores de risco para DRC incluem histórico familiar de doença renal (por exemplo, doença renal mediada por APOL1, comum em indivíduos com ascendência da África Ocidental), IRA anterior, doença renal relacionada à gestação (por exemplo, pré-eclâmpsia), malignidade, distúrbios autoimunes (lúpus eritematoso sistêmico, vasculite), indivíduos nascidos com baixo peso ou pré-termo, uropatia obstrutiva, nefrolitíase recorrente e anomalias congênitas do rim e do trato urinário (CAKUT) (ver Figura 1)^3^. Os determinantes sociais da saúde influenciam significativamente o risco de desenvolver DRC, tanto em nível individual quanto nacional. Nos LICs e LMICs, acredita-se que o estresse térmico possa causar DRC de etiologia desconhecida em trabalhadores rurais, uma causa mundial de DRC cada vez mais reconhecida^17^. Além disso, envenenamentos, toxinas ambientais, medicamentos tradicionais e infecções (hepatite viral B ou C, HIV e parasitas) merecem ser considerados como grupos de risco, especialmente em áreas endêmicas^18,19^.

**Figura 1:** Fatores de risco para doença renal crônica.

## Como Podemos Avaliar a Saúde Renal?

Conceitualmente, existem três níveis de prevenção da DRC. A prevenção primária reduz a incidência de DRC por meio do tratamento dos fatores de risco; a prevenção secundária reduz a progressão e as complicações naqueles com DRC detectada; e a prevenção terciária melhora os desfechos em pacientes com insuficiência renal, promovendo melhorias no manejo, como vacinação aprimorada ou fornecimento ideal de diálise^20^. As estratégias de prevenção primária e secundária podem utilizar as 8 regras de ouro para a promoção da saúde renal, que incluem: dieta saudável, hidratação adequada, atividade física, monitoramento e controle da pressão arterial, monitoramento e controle glicêmico, evitar o uso de nicotina, evitar o uso regular de anti-inflamatórios não esteroides e testes direcionados para indivíduos com fatores de risco^21^. Cinco dessas recomendações são idênticas às regras do “*Life’s Essential 8*”, que visam à melhoria e à manutenção da saúde cardiovascular, incluindo também peso e sono adequados e manejo de lipídios^22^. A detecção precoce se concentra na prevenção secundária da DRC, que envolve a proteção da saúde renal e a redução do risco cardiovascular.

### Seus Rins Estão Saudáveis?

Mundialmente, a detecção precoce da DRC é rara, irregular e ainda menos provável de ocorrer em LICs ou LMICs. Atualmente, somente três países possuem um programa nacional voltado à testagem ativa de populações em risco para DRC, e outros 17 países testam ativamente populações em risco durante exames de saúde de rotina^23^. Mesmo em HICs, a albuminúria não é avaliada em mais da metade dos indivíduos com DM2 e/ou hipertensão^24,25,26^. Surpreendentemente, naqueles com função renal reduzida documentada, o diagnóstico de DRC geralmente não é realizado. Um estudo em HICs revelou ausência de diagnóstico de DRC entre 62–96% da população com evidência laboratorial de DRC estágio G3^27^.

Recomendamos que os profissionais de saúde realizem os seguintes exames em todos os grupos de risco, a fim de avaliar a saúde renal (Figura 2):

a) Medições da pressão arterial, uma vez que a hipertensão é o fator de risco mais prevalente para doença renal em todo o mundo^3,28,29^.
b) Índice de massa corporal (IMC), já que a obesidade está epidemiologicamente associada ao risco de DRC, de forma indireta por meio de DM2 e hipertensão e como um fator de risco independente. A adiposidade visceral contribui para a microinflamação por monócitos e o risco cardiometabólico renal^3,28,29^.
c) Hemoglobina glicada, glicemia de jejum ou teste aleatório de glicose, visto que o DM2 é um fator de risco comum para doença renal^3,28,29^.
d) A avaliação da função renal por meio da creatinina sérica para estimar a TFG (TFGe) é recomendada em todos os contextos^3^. A TFG deve ser estimada com uma equação validada, livre de ajustes para raça e adequada ao país ou região e à faixa etária^3^. Em geral, uma TFGe < 60 mL/min/1,73m^2^ é o limite para DRC em adultos e crianças, embora um limite de < 90 mL/min/1,73m^2^ possa ser sinalizado como “baixo” em crianças e adolescentes com idade superior a 2 anos^3^. Uma limitação da TFGe baseada na creatinina é o fato de a creatinina também ser um biomarcador de nutrição e massa muscular. Portanto, estados de desnutrição e fragilidade superestimam a função renal^3,30^. Dessa forma, a TFGe que combina creatinina sérica e cistatina C é geralmente mais precisa do que o uso isolado de qualquer um dos biomarcadores na maioria dos contextos clínicos. No entanto, a viabilidade do uso da cistatina C é limitada principalmente aos HICs devido à disponibilidade do ensaio e ao custo da creatinina^3,30,31^.
e) Teste de lesão renal (albuminúria). Em adultos e crianças, recomenda-se uma amostra da primeira urina da manhã para avaliação da albuminúria^3^. Em adultos, a relação albumina-creatinina urinária (RACu) quantitativa é preferível, por ser o teste mais sensível^3^. Importante ressaltar que a albumina urinária encontra-se em processo de padronização dos métodos analíticos, o que, em última instância, deve facilitar a padronização mundial da RACu^32^. Em crianças, tanto a relação proteína-creatinina (RPCu) quanto a RACu devem ser testadas com o objetivo de avaliar a proteinúria tubular^3^. O teste semiquantitativo de albuminúria permite flexibilidade para a realização de testes no local de atendimento (“*point-of-care*”) ou em ambiente domiciliar^33^. Testes de triagem semiquantitativos ou qualitativos devem apresentar positividade em >85% dos indivíduos com RACu quantitativa de 30 mg/g ou mais para serem considerados úteis^34^. Em ambientes com recursos limitados, pode-se utilizar o teste de tira reagente para proteína na urina, com um limite de proteinúria igual ou superior a + 2, visando reduzir os resultados falso-positivos para repetição do teste confirmatório^35^.

**Figura 2:** Estrutura conceitual para testes, estratificação de risco e programa de tratamento da doença renal crônica (DRC), ver referência^30^.

Em populações específicas, podem ser considerados os seguintes testes:

f) Exames para hematúria – conhecida atualmente como fator de risco esquecido em diretrizes recentes de prática clínica – são particularmente importantes para aqueles com risco de doença glomerular, especialmente nefropatia por IgA^36^.
g) É importante a realização de exames de imagem iniciais (basais) em grupos com sinais ou sintomas sugestivos de anormalidades estruturais (por exemplo, dor e hematúria), com o objetivo de avaliar massas renais, cistos, cálculos, hidronefrose ou retenção urinária. A ultrassonografia pré-natal pode detectar hidronefrose e outras CAKUT.
h) Com a crescente disponibilidade de testes genéticos, o teste em cascata para DRC familiar é indicado quando há um risco genético conhecido de doença renal^37^.
i) Em indivíduos com risco ocupacional de desenvolver doença renal, o exame renal deve ser oferecido como parte dos programas de saúde ocupacional.
j) Indivíduos doadores de rins devem ser incluídos em programas de vigilância pós-doação para avaliar a saúde renal em longo prazo^38^.

## Benefícios Potenciais da Detecção Precoce

A triagem para DRC está de acordo com muitos dos princípios de Wilson-Jungner da Organização Mundial da Saúde. A DRC em estágio inicial é assintomática, e existem tratamentos eficazes estabelecidos, incluindo modificação do estilo de vida, cuidados interdisciplinares e intervenções farmacológicas^2,3,30,35^. Medicamentos essenciais da OMS que melhoram os desfechos da DRC, incluindo inibidores da ECA, bloqueadores dos receptores de angiotensina, estatinas e inibidores do cotransportador de sódio-glicose tipo 2 (SGLT2i), devem estar amplamente disponíveis^2,39^. Estima-se que os SGLT2i, isoladamente, reduzam o risco de progressão da DRC em 37% em indivíduos com e sem diabetes^40^. Para uma pessoa de 50 anos com albuminúria e DRC não diabética, esses resultados poderiam estender o período de função renal saudável de 9,6 anos para 17 anos^41^. Esses medicamentos essenciais reduzem a progressão para estágios mais avançados da DRC e limitam a hospitalização por causas cardiovasculares, proporcionando custo-benefício em curto prazo, especialmente nos países de baixa renda. Quando disponíveis e acessíveis, a gama de novos medicamentos transformadores de paradigmas destinados a retardar a progressão da DRC também inclui antagonistas do receptor do peptídeo-1 semelhante ao glucagon, antagonistas dos receptores mineralocorticoides não esteroidais, antagonistas dos receptores de endotelina e medicamentos específicos modificadores da doença (como inibidores do complemento) que anunciam uma nova e promissora era para a nefrologia.

Considerando os custos significativos de saúde associados à DRC, especialmente de hospitalização e insuficiência renal, medidas preventivas eficazes oferecem benefícios econômicos evidentes tanto para países de alta quanto de baixa renda. A DRC impõe custos expressivos aos indivíduos, suas famílias, sistemas de saúde e governos em todo o mundo. Nos Estados Unidos, a DRC representa um custo anual de mais de US$ 85 bilhões para o sistema Medicare^13^. Em muitos países de alta e média renda, 2 a 4% do orçamento destinado à saúde é gasto apenas com os cuidados com a insuficiência renal. Na Europa, os custos de saúde associados à DRC superam os associados ao câncer ou ao diabetes^42^. A redução do ônus do tratamento renal em todo o mundo também trará efeitos ambientais significativos, uma vez que proporcionará economia de água e reduzirá o desperdício de plástico, especialmente associado à diálise^43^. Em nível individual, os custos da DRC são frequentemente calamitosos, especialmente em LICs e LMICs, onde o ônus do pagamento recai em grande parte sobre o próprio paciente. Somente 13% dos LICs e 19% dos LMICs cobrem os custos da TRS para adultos^15^. A DRC leva 188 milhões de pessoas em países de baixa e média-baixa renda a enfrentarem anualmente despesas catastróficas com a saúde^44^.

O limite da razão de custo-efetividade incremental (ICER, *Incremental Cost Effectiveness Ratio*) mais amplamente citado e estudado para avaliar a triagem é de US$ <50.000 por ano de vida ajustado à qualidade (QALY)^45^. Caso a prevalência de DRC seja elevada, uma estratégia de triagem em toda a população deve ser considerada em HICs^33,46^. Por exemplo, nos Estados Unidos, um modelo recente de simulação de Markov para triagem populacional de DRC, que incluiu o tratamento adequado com SGLT2i somado ao tratamento padrão com inibidores da ECA ou bloqueadores dos receptores de angiotensina para adultos de 35 a 75 anos com albuminúria, concluiu que a triagem para identificar DRC seria custo-efetiva^46^. Além disso, uma análise de uma triagem semiquantitativa de albuminúria da população geral, realizada em ambiente domiciliar na Holanda, também demonstrou ser custo-efetiva^33^. A detecção de casos para identificar a DRC em grupos de risco mais elevado, em vez da triagem em massa ou da população geral, reduzirá os custos e outros possíveis danos, ao mesmo tempo em que aumentará a taxa de verdadeiros positivos nos testes de triagem^3,35,45^. Um limite alternativo de ICER proposto pela OMS é de <1-3 vezes a razão do produto interno bruto per capita por QALY, que pode ser utilizado para avaliar abordagens de detecção de casos em LIC e LMIC^45^. Os testes recomendados para a detecção da doença renal são de baixo custo e minimamente invasivos, facilitando sua administração em diversos contextos.

Os testes básicos de TFGe e RAC urinária estão amplamente disponíveis, e o uso do teste de urina com fita reagente nos casos em que o teste quantitativo de proteinúria não está disponível ou é inacessível reduzirá drasticamente os custos dos testes^31^.

Se associada a intervenções eficazes, a identificação precoce de indivíduos com doença renal beneficiará o indivíduo, o sistema de saúde, os governos e a economia^44^. Os benefícios à saúde e à qualidade de vida do indivíduo resultariam em maior produtividade, especialmente para os jovens que possuem mais anos de atividade profissional pela frente, e em melhorias no desenvolvimento/educação de crianças e jovens adultos. Os indivíduos enfrentariam menos despesas catastróficas com a saúde, os governos e os sistemas de saúde economizariam dinheiro não apenas nos cuidados com a DRC, mas também nos custos com doenças cardiovasculares, e as economias se beneficiariam de uma maior participação no mercado de trabalho. Trata-se de algo particularmente crucial para os países de baixa renda, onde o ônus da DRC recai sobre os governos e os indivíduos que não podem arcar com os custos dos cuidados renais.

## Desafios e Soluções Para a Implementação

As barreiras estruturais para a ampla identificação e o tratamento de pessoas com DRC incluem custo, confiabilidade dos testes e falta de sistemas de informações de saúde para rastrear o ônus da DRC. Essas são sustentadas pela falta de políticas governamentais e de saúde relevantes, baixo conhecimento e implementação por parte dos profissionais de saúde, baixa percepção do risco de doença renal na população em geral e baixa conscientização da DRC por parte dos pacientes.

As soluções para a implementação de intervenções eficazes incluem vincular a identificação da DRC aos programas de triagem existentes, educar o público e os profissionais da atenção primária e aproveitar os programas de defesa conjunta de organizações não governamentais (ONGs) para que as agendas de políticas de saúde se concentrem na doença renal. Qualquer solução deve equilibrar os potenciais benefícios e danos dos programas de triagem e detecção de casos. As implicações éticas a serem consideradas incluem a disponibilidade de recursos (como profissionais de saúde e medicamentos), a viabilidade econômica dos testes e do tratamento, falsos positivos ou negativos e a ansiedade dos pacientes e de suas famílias^47^.

Os programas de triagem e detecção de casos exigem capacidade da força de trabalho, sistemas de informação em saúde, equipamentos de teste confiáveis e acesso equitativo a cuidados médicos, medicamentos, vacinas e tecnologias em saúde.

A atenção primária está na linha de frente da batalha para proteger a saúde renal, principalmente em países de baixa renda e países de renda média-baixa. A reduzida força de trabalho em nefrologia, com uma prevalência global mediana de 11,8 nefrologistas por milhão de habitantes e uma discrepância de 80 vezes entre LICs e HICs, é inadequada para a detecção e o manejo da grande maioria dos casos de DRC^23^. Assim como em outras doenças crônicas, os médicos da atenção primária e outros profissionais de saúde da linha de frente são fundamentais para a detecção precoce da DRC^48^. Os testes devem ser acessíveis, simples e práticos, sendo que o teste *point of care* de creatinina e as fitas reagentes para análise de urina são úteis em locais com recursos limitados^31^.

Esforços educacionais direcionados aos médicos da atenção primária são fundamentais para integrar a detecção da DRC à rotina de atendimento, apesar da limitação de tempo e recursos^49,50,51^. O suporte automatizado a decisões clínicas pode aproveitar os registros de saúde eletrônicos para identificar pessoas com DRC ou com alto risco de DRC e recomendar aos médicos as ações adequadas (Figura 2).

Atualmente, poucos países dispõem de registros de DRC, o que limita nossa capacidade de ressaltar o ônus da doença perante os governos. O conhecimento sobre o ônus da DRC auxilia na priorização das necessidades de saúde renal, que devem se expandir progressivamente de modo a abranger todo o espectro do cuidado renal^52^. Uma pesquisa global revelou que apenas um quarto dos países (41/162) tinha uma estratégia nacional específica para a DRC e menos de um terço (48/162) reconhecia a DRC como uma prioridade de saúde pública^23^. O reconhecimento da DRC pela OMS como um dos principais fatores de mortalidade por DNTs seria eficaz para aumentar a conscientização, melhorar a vigilância e o monitoramento locais visando à implementação de diretrizes de práticas clínicas, além de melhorar a alocação de recursos^2^.

Os programas para a detecção precoce da DRC exigirão ampla coordenação e envolvimento das partes interessadas, incluindo governos, sistemas de saúde e seguradoras. Organizações renais internacionais e nacionais, como a *International Society of Nephrology* (ISN), já defendem junto à OMS e a governos individuais a priorização da doença renal. Devemos dar continuidade a esse trabalho, colaborando para agilizar o planejamento e a implementação do programa de detecção precoce. A integração com programas comunitários existentes (por exemplo, prevenção de doenças cardiovasculares) em LMICs e HICs pode reduzir os custos e maximizar a eficiência. Tais programas precisarão ser adaptados ao contexto local e podem ser realizados em uma variedade de ambientes, como consultórios médicos individuais, hospitais, unidades de saúde regionais ou nacionais ou como alcance a comunidades rurais. Dependendo das regulamentações e dos recursos locais, a triagem e a detecção de casos também podem ocorrer fora de ambientes médicos, como prefeituras, igrejas ou feiras. Voluntários locais também podem auxiliar nos esforços de triagem e detecção de casos dentro da comunidade.

Paralelamente à reorientação da prática clínica dos profissionais de saúde para a detecção oportuna da DRC, devemos nos concentrar na educação da população em geral sobre o risco percebido e nas atividades de promoção da saúde e programas educacionais destinados a conscientizar e empoderar os pacientes. A conscientização sobre a doença renal é baixa, sendo que 9 em cada 10 pessoas com DRC desconhecem que são afetadas^53^. A doença renal é pouco abordada na mídia leiga, e uma análise revelou que ela foi 11 vezes menos representada em comparação à causa real do óbito^54^. Diversas organizações nacionais e internacionais desenvolveram questionários para o público em geral sobre o risco de doença renal. Esse fato foi apoiado por um estudo regional que demonstrou que pacientes socialmente vulneráveis com hipertensão não compreendem os riscos renais a que estão expostos^21,55,56,57^. A educação, tanto online quanto presencial, voltada para os profissionais de saúde, pode melhorar a alfabetização em saúde do consumidor. A ativação, o comprometimento e a tomada de decisão compartilhada do paciente são impactos posteriores da conscientização. A conscientização sobre a DRC é diferenciada, incluindo a detecção e a estratificação de risco para informar e empoderar, em vez de amedrontar, quanto ao momento e à extensão das intervenções (ver Quadro 1)^4,27,57^. Encontrar o equilíbrio adequado otimizará a autoeficácia e o envolvimento de pacientes, familiares e cuidadores.

**Quadro 1:** Seus rins estão saudáveis? perspectivas pessoais sobre conscientização, detecção e tratamento da DRC com base na literatura ^4,57^

Na verdade, eu não entendia muito bem por que ninguém tinha me dado todas as informações sobre o que eu tinha, de uma forma que eu pudesse dizer: “Bem, isso é o que eu tenho [DRC] e é por isso que eu tenho”.
Eles [os médicos] podem responder a essas perguntas [sobre saúde renal], (...) mas é tudo muito técnico.
Eu não sabia o que significava [DRC], então não podia realmente compartilhar com outras pessoas.
Talvez eu não saiba quais são meus índices [de saúde renal], mas sei quais são os exames e sei que já os fiz antes.
Bem, deixe-me dizer o seguinte: Agora eu estou bem ciente da importância dos rins e de quais são os problemas aqui. E eu certamente consideraria... Quando eu for ao médico, diria a ele: “Escute, o senhor fez os exames de sangue. Mas como estão os meus rins? Quais são os resultados?”
Eu sei que eles fizeram exames de urina no passado, e sei que havia proteína e açúcar na minha urina.
Eu saí de nunca tomar um comprimido para tomar 22 comprimidos. O que está acontecendo aqui? Eu não sabia o que eram. Mas eu apenas os numerei e isso me ajudou muito porque percebi o que estava acontecendo, mas alguns deles, toda vez que eu ia lá [ao médico], eu recebia outro comprimido. Eu sabia que tinha de tomá-lo porque eles sabiam o que estavam fazendo, os médicos que eu consultava.
Isso [DRC] é algo novo, então, imediatamente, eu estava pensando que era só mais uma coisa com que me preocupar. Mas depois me senti um pouco empoderado e como se eu realmente quisesse me antecipar a isso. Sinto que quero ter uma conversa com meu médico da atenção primária.
O que mais me interessa é saber o que está acontecendo, por que está acontecendo e o que posso fazer para desacelerar isso [DRC]?

## Conclusão: um Chamado à Ação

Convocamos todos os profissionais de saúde a avaliar a saúde renal de seus pacientes com risco de doença renal. Em paralelo, devemos trabalhar em conjunto com as organizações de saúde pública no sentido de melhorar a percepção da população em geral sobre o risco de desenvolver doença renal e capacitar os indivíduos em risco a buscar acompanhamento da saúde renal. Para viabilizar essa mudança, precisamos atuar junto aos sistemas de saúde, aos governos e à OMS para priorizar a doença renal e criar programas eficazes e eficientes de detecção precoce da doença renal. Só então os benefícios da mudança de paradigma decorrentes das alterações no estilo de vida e dos tratamentos farmacológicos se traduzirão em melhor saúde renal e geral para as pessoas em todo o mundo.

## Data Availability

Não há novos dados associados a este artigo.
